# Deciphering the causal landscape: genetic insights into sporadic vestibular schwannoma risk factors through Mendelian Randomization

**DOI:** 10.1007/s12672-024-01644-3

**Published:** 2024-12-02

**Authors:** Yuyang Liu, Hui Feng, Hengchao Ma, Jing Li, Yang Yu, Hua Zhao, Xiaoguang Wang, Yun Li, Jun Zhang, Qi Liu

**Affiliations:** 1 Department of Neurosurgery, 920th Hospital of Joint Logistics Support Force, Kunming, 650032 China; 2 Department of Zhantansi Outpatient, Jingzhong Medical District of Chinese People’s Liberation Army General Hospital, Beijing, 100034 China; 3 Medical School of Chinese People’s Liberation Army, Beijing, 100853 China; 4 Department of Neurosurgery, Chinese People’s Liberation Army General Hospital, Beijing, 100853 China; 5 Faculty of Hepato-Pancreato-Biliary Surgery, The First Medical Center of Chinese People’s Liberation Army General Hospital, Beijing, 100853 China

**Keywords:** Vestibular schwannoma, Mendelian Randomization, Genetic risk factors, Genome-wide association studies, Epidemiology

## Abstract

**Background:**

Sporadic vestibular schwannoma, a benign tumor affecting the vestibulocochlear nerve, poses significant health challenges due to its impact on hearing, balance, and facial nerve function. Despite known associations with genetic mutations and environmental factors, the causality between potential risk factors and sporadic vestibular schwannoma remains underexplored.

**Objective:**

This study aims to investigate the causal effects of various genetically predicted risk factors on sporadic vestibular schwannoma utilizing a Two-Sample Mendelian Randomization (MR) approach to enhance understanding of its etiology and inform prevention strategies.

**Methods:**

Leveraging data from genome-wide association studies (GWAS), we analyzed 29 risk factors across five categories: related diseases, lifestyle habits, nutritional status, learning ability, and laboratory indicators. The MR analysis employed instrumental variables (IVs) derived from single nucleotide polymorphisms (SNPs) to assess causal relationships, overcoming traditional observational study limitations.

**Results:**

Our findings highlight significant associations between sporadic vestibular schwannoma and factors such as ovarian cancer, uterine fibroids and lifestyle habits including dietary intake and alcohol consumption. Notably, higher educational attainment and specific laboratory indicators like high-density lipoprotein (HDL) cholesterol levels were linked to altered disease risk. These results suggest a multifaceted etiology involving hormonal, cardiovascular, gastrointestinal, immune, and metabolic pathways.

**Conclusion:**

This comprehensive MR study provides novel insights into the diverse risk factors contributing to sporadic vestibular schwannoma, emphasizing the role of genetic predispositions, hormonal influences, and lifestyle choices in its development. The associations identified underscore the need for a multidisciplinary research approach and targeted public health strategies to mitigate sporadic vestibular schwannoma risk. Further research into the underlying mechanisms of these associations is crucial for developing effective interventions and improving patient outcomes.

## Introduction

Vestibular schwannoma, also known as an acoustic neuroma, is a benign intracranial tumor arising from the Schwann cells covering the vestibular nerve [1]. Despite its non-malignant nature, vestibular schwannoma can result in significant morbidity due to its location and its potential effects on hearing, balance, and facial nerve function [2, 3]. Microsurgery represents the primary treatment modality [4]. The etiology of sporadic vestibular schwannoma remains incompletely understood, with identified risk factors including genetic predispositions, such as mutations in the *NF2* gene [5], radiation to the head and neck [6, 7], and environmental influences [8–10]. Despite these advances, establishing causality between risk factors and disease outcomes remains challenging due to the inherent limitations of observational studies, such as confounding factors and reverse causation.

To address these challenges, Mendelian Randomization (MR) provides a potent tool in epidemiological research. This method utilizes genetic variants, such as single nucleotide polymorphisms (SNPs), as instrumental variables (IVs) to enable the assessment of causal relationships, thereby overcoming the limitations of traditional observational studies [11–13], including confounding by socio-economic and lifestyle factors. By leveraging data from genome-wide association studies (GWAS), MR enables researchers to infer causal effects of exposures on disease outcomes, thus significantly enhancing our understanding of disease mechanisms and heritability [14].

In this study, a comprehensive Two-Sample MR analysis was conducted to investigate the causal effects of 29 genetically predicted potential risk factors on sporadic vestibular schwannoma. These risk factors encompass related diseases, lifestyle habits, nutritional status, learning abilities, and laboratory indicators. The primary objective of this research is to furnish a detailed overview of the diverse risk factors for sporadic vestibular schwannoma and to provide novel insights into its etiology. By elucidating the causal relationships between these factors and sporadic vestibular schwannoma, this study aims to contribute significantly to the field of neuro-oncology and to inform public health strategies. The findings of this research possess the potential to significantly enhance our understanding of the etiology of sporadic vestibular schwannoma and to guide future efforts in disease prevention and management.

## Materials and methods

### MR design

In this research, MR was employed to explore the associations between various risk factors and sporadic vestibular schwannoma. This study identified and analyzed 29 primary risk factors, categorized into five groups: related diseases, lifestyle habits, nutritional status, learning abilities, and laboratory indicators. SNPs linked to these risk factors served as IVs in the analysis. This approach facilitated a more comprehensive understanding of the potential genetic and environmental contributors to sporadic vestibular schwannoma. A valid IV in MR must satisfy three essential criteria: (1) Relevance Criterion: Each IV is required to demonstrate a robust and consistent relationship with the exposure, ensuring significant impact on the exposure factor. (2) Independence Criterion: The IV should not be associated with any confounders influencing the exposure-outcome relationship, crucial for credible causal conclusions. (3) Exclusion Restriction: The effect of IV on the outcome must occur solely through the exposure, without alternative pathways. The phenomenon of horizontal pleiotropy, wherein an IV influences the outcome via pathways unrelated to the exposure, presents a significant challenge to meeting this criterion.

To enhance the robustness of the MR analysis, genetic instruments were selected based on stringent criteria that incorporated linkage disequilibrium (LD) considerations and genome-wide significance levels. Using the TwoSampleMR package in R, genetic variants were identified with a genome-wide significance threshold of *p* < 5 × 10^–8^. An LD clumping procedure was conducted to ensure SNP independence, with an r^2^ threshold of 0.001 and a physical distance criterion of 10,000 kilobases (kb) around each SNP, thereby effectively filtering out SNPs in high LD and retaining independent exposure predictors. Additionally, a critical filter was applied to the minor allele frequency (MAF), excluding SNPs with a MAF less than 0.01. This criterion was crucial for minimizing potential biases introduced by rare variants, thus improving the generalizability and reliability of the genetic instruments. The instrumental strength of each genetic variant was quantified using the F-statistic, defined as F = R^2^ (n − 2) (1 − R^2^), where R^2^ represents the proportion of phenotypic variance explained by the genetic variant, and n signifies the sample size. A threshold of F > 10 was established to identify robust instruments, thereby mitigating the risk of weak instrument bias [15]. This meticulous selection process, encompassing considerations for LD, MAF, and instrumental strength, played a pivotal role in reducing potential biases from correlated genetic variants and weak instruments, ensuring that the genetic instruments used possessed substantial strength and independence for valid MR analysis findings.

The Inverse Variance Weighted (IVW) method was employed as the primary analytical tool in this MR study to estimate the overall effect size, in alignment with the recommendations of Burgess et al. [16]. This method calculates the influence of each genetic variant on disease risk, proportional to its effect on the exposure, by utilizing the Wald ratio within the IVW framework. A random-effects inverse variance meta-analysis was then conducted to derive a comprehensive summary effect estimate. To ensure the integrity and reliability of the findings, the validity of the underlying MR assumptions was rigorously examined through a series of sensitivity analyses.

In addition to the IVW method, complementary approaches such as MR-Egger [17] and the weighted median method [18] were employed, utilizing the R TwoSampleMR package [19] for these analyses. SNP heterogeneity was assessed using Cochran’s Q test, with a *p* > 0.05 considered indicative of homogeneity [20].

**Fig. 1:**
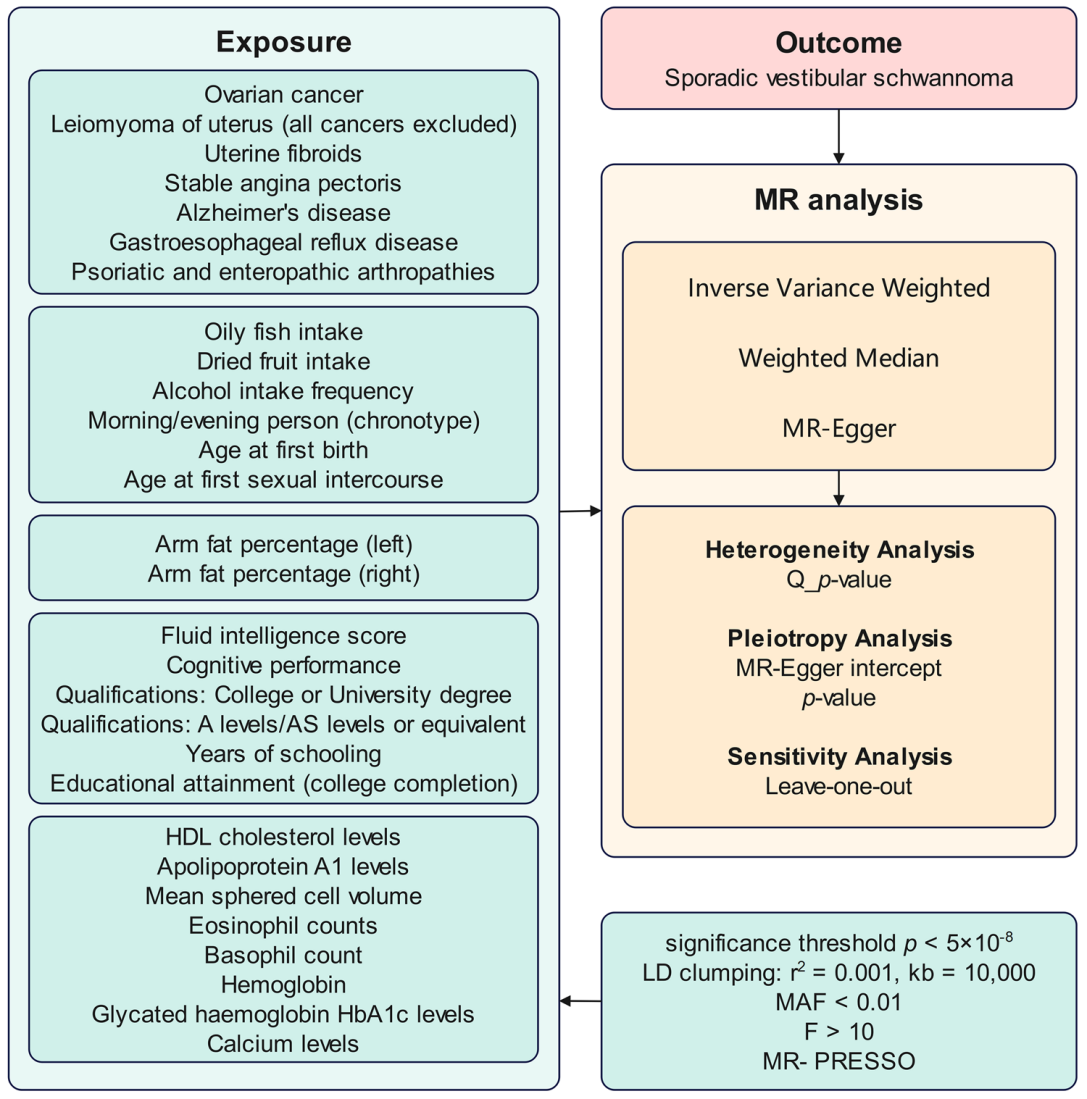
workflow of MR analysis

The MR-Pleiotropy RESidual Sum and Outlier (PRESSO) global test and the MR-Egger intercept were used to identify outliers and horizontal pleiotropy [21]. The MR-Egger intercept was utilized to facilitate the quantification of the average pleiotropic effect. The MR-PRESSO test was applied to correct for horizontal pleiotropy and to detect significant distortions in causal estimates. Additionally, a leave-one-out analysis was conducted to evaluate the impact of individual SNPs on the overall results (Fig. 1).

### Genetic instrument selection

For the genetic analysis of the 29 risk factors, GWAS involving individuals of European ancestry were selected as the primary data sources (Table 1). Genetic instruments for Ovarian cancer were extracted from the Ovarian Cancer Association Consortium (OCAC) [22]. GWAS summary statistics for Oily fish intake, Dried fruit intake, Qualifications: College or University degree, Alcohol intake frequency, Morning/evening person (chronotype) were obtained from the MRC Integrative Epidemiology Unit (MRC-IEU) consortium [23]. IVs for Alzheimer’s disease [24] were chosen from Alzheimer Disease Genetics Consortium (ADGC), European Alzheimer’s Disease Initiative (EADI), Cohorts for Heart and Aging Research in Genomic Epidemiology Consortium (CHARGE), Genetic and Environmental Risk in AD/Defining Genetic, Polygenic and Environmental Risk for Alzheimer’s Disease Consortium (GERAD/PERADES). Genetic instruments for Qualifications: A levels/AS levels or equivalent, Arm fat percentage (left), Arm fat percentage (right), Fluid intelligence score was selected from Neale Lab (http://www.nealelab.is). IVs for Years of schooling, Cognitive performance were chosen from Social Science Genetic Association Consortium (SSGAC) [25]. GWAS summary statistics for Leiomyoma of uterus (all cancers excluded), Psoriatic and enteropathic arthropathies were received from FinnGen [26]. For Uterine fibroids [27], Stable angina pectoris [27], Glycated haemoglobin HbA1c levels [28], Calcium levels [28], Basophil count [28], Gastroesophageal reflux disease [29], Educational attainment (college completion) [30], high-density lipoprotein (HDL) cholesterol levels [31], Apolipoprotein A1 levels [31], Mean sphered cell volume [31], Eosinophil counts [31], Hemoglobin [32], Age at first birth [33], Age at first sexual intercourse [33] were selected from associated GWAS studies.

**Table 1:** Characteristics of the GWAS summary data

Exposure	Reference	Sample Size	SNPs	F	Source
Related diseases					
Ovarian cancer	[22]	66,450	9	62.3	OCAC
Leiomyoma of uterus (all cancers excluded)	[26]	107,042	15	71.5	FinnGen
Uterine fibroids	[27]	258,718	40	64.7	PMID
Stable angina pectoris	[27]	343,026	41	54.3	PMID
Alzheimer’s disease	[24]	63,926	18	137.2	PMID
Gastroesophageal reflux disease	[29]	602,604	66	39.0	PMID
Psoriatic and enteropathic arthropathies	[26]	174,613	4	112.2	FinnGen
Lifestyle habits					
Oily fish intake	[23]	460,443	51	45.2	MRC-IEU
Dried fruit intake	[23]	421,764	35	40.3	MRC-IEU
Alcohol intake frequency	[23]	462,346	89	54.0	MRC-IEU
Morning/evening person (chronotype)	[23]	413,343	138	46.7	MRC-IEU
Age at first birth	[33]	418,758	53	39.3	PMID
Age at first sexual intercourse	[33]	182,791	47	40.9	PMID
Nutritional Status					
Arm fat percentage (left)	[34]	331,198	231	53.5	Neale Lab
Arm fat percentage (right)	[34]	331,249	213	54.5	Neale Lab
Learning ability					
Fluid intelligence score	[34]	108,818	40	36.7	Neale Lab
Cognitive performance	[25]	257,841	117	43.7	SSGAC
Qualifications: college or University degree	[23]	458,079	217	47.2	MRC-IEU
Qualifications: a levels/AS levels or equivalent	[34]	334,070	53	38.7	Neale Lab
Years of schooling	[25]	766,345	263	49.2	SSGAC
Educational attainment (college completion)	[30]	470,941	182	45.8	PMID
Laboratory indicators					
HDL cholesterol levels	[31]	400,754	305	240.0	PMID
Apolipoprotein A1 levels	[31]	398,508	249	211.5	PMID
Mean sphered cell volume	[31]	437,736	349	172.5	PMID
Eosinophil counts	[31]	440,275	317	130.2	PMID
Basophil count	[28]	395,949	75	86.5	PMID
Hemoglobin	[32]	408,112	307	103.0	PMID
Glycated haemoglobin HbA1c levels	[28]	389,889	305	130.6	PMID
Calcium levels	[28]	357,831	186	87.9	PMID

### Sporadic vestibular schwannoma data source

The summary data for sporadic vestibular schwannoma in this study were sourced from a GWAS study conducted by Sadler KV [35], which comprised 911 cases and 5500 controls of British ancestry. All case samples were identified via the highly specialized *NF2* genetic screening service located in the north-west region of England, UK. These samples were gathered from both the Manchester Centre for Genomic Medicine and the Salford Royal Foundation Trust. All control samples were sourced from the UK Biobank (UKBB) initiative. The UKBB stands as a comprehensive biomedical repository, hosting genetic and extensive phenotypic information for approximately half a million individuals residing in the UK, aged between 49 and 60 [36]. Every selected GWAS received ethical clearance, with participants providing their informed consent.

### Software

All statistical analyses in this study were conducted using R software (version 4.3.0). This version was selected for its advanced features and robust computational power, essential for effectively conducting complex statistical computations inherent in MR studies and other sophisticated epidemiological analyses. This choice aligns with the requirements for precision and accuracy in managing large datasets and intricate statistical models. The comprehensive range of packages and tools in R, particularly those developed for genetic and epidemiological research, provided the necessary support for the extensive data analysis and interpretation.

## Results

**Fig. 2:**
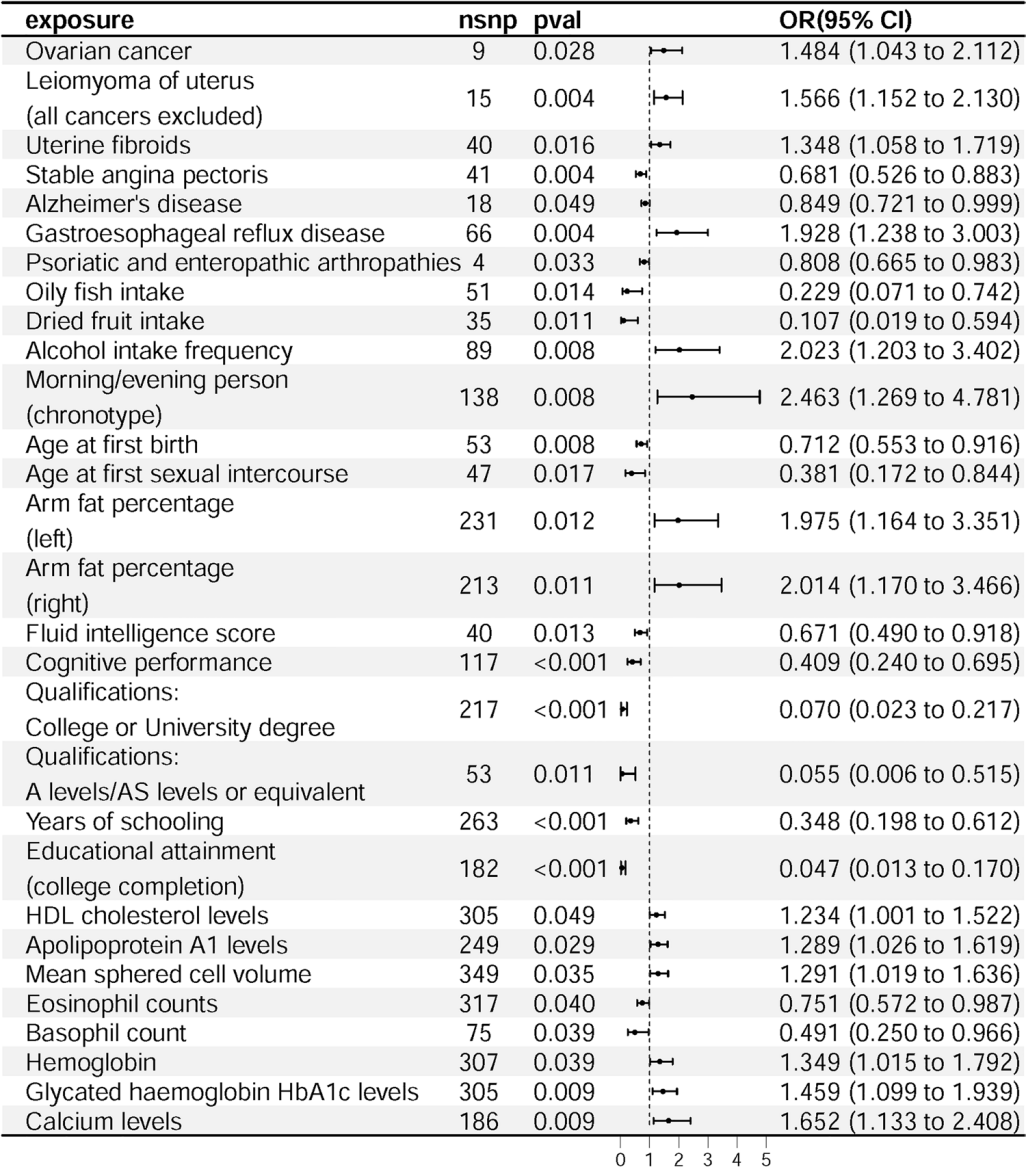
Forest plot of OR and 95% CI (lines). Overview impact calculations of MR indicating relationships between sporadic vestibular schwannoma and risk factors

Univariate MR analyses identified several exposure factors significantly associated with an increased risk of sporadic vestibular schwannoma. Among these, risk factors include Ovarian cancer (IVW: OR = 1.484, *p* = 0.028; MR-Egger: OR = 3.131, *p* = 0.039), Leiomyoma of uterus (all cancers excluded) (IVW: OR = 1.566, *p* = 0.004), Uterine fibroids (IVW: OR = 1.348, *p* = 0.016; Weighted Median: OR = 1.450, *p* = 0.046), Gastroesophageal reflux disease (IVW: OR = 1.928, *p* = 0.004; Weighted Median: OR = 1.919, *p* = 0.049), Alcohol intake frequency (IVW: OR = 2.023, *p* = 0.008), Morning/evening person (chronotype) (IVW: OR = 2.463, *p* = 0.008), Arm fat percentage (left) (IVW: OR = 1.975, *p* = 0.012), Arm fat percentage (right) (IVW: OR = 2.014, *p* = 0.011), HDL cholesterol levels (IVW: OR = 1.234, *p* = 0.049), Apolipoprotein A1 levels (IVW: OR = 1.289, *p* = 0.029), Mean sphered cell volume (IVW: OR = 1.291, *p* = 0.035), Hemoglobin (IVW: OR = 1.349, *p* = 0.039; MR-Egger: OR = 1.783, *p* = 0.030), Glycated haemoglobin HbA1c levels (IVW: OR = 1.459, *p* = 0.009), Calcium levels (IVW: OR = 1.652, *p* = 0.009; Weighted Median: OR = 1.934, *p* = 0.032). Identified protective factors inversely associated with the risk of sporadic vestibular schwannoma include Stable angina pectoris (IVW: OR = 0.681, *p* = 0.004), Alzheimer’s disease (IVW: OR = 0.849, *p* = 0.049), Psoriatic and enteropathic arthropathies (IVW: OR = 0.808, *p* = 0.033), Oily fish intake (IVW: OR = 0.229, *p* = 0.014), Dried fruit intake (IVW: OR = 0.107, *p* = 0.011; Weighted Median: OR = 0.054, *p* = 0.018), Age at first birth (IVW: OR = 0.712, *p* = 0.008), Age at first sexual intercourse (IVW: OR = 0.381, *p* = 0.017), Fluid intelligence score (IVW: OR = 0.671, *p* = 0.013; Weighted Median: OR = 0.638, *p* = 0.034), Cognitive performance (IVW: OR = 0.409, *p* < 0.001; Weighted Median: OR = 0.317, *p* = 0.003), Qualifications: College or University degree (IVW: OR = 0.070, *p* < 0.001; Weighted Median: OR = 0.050, *p* < 0.001), Qualifications: A levels/AS levels or equivalent (IVW: OR = 0.055, *p* = 0.011; Weighted Median: OR = 0.011, *p* = 0.006), Years of schooling (IVW: OR = 0.348, *p* < 0.001; Weighted Median: OR = 0.317, *p* = 0.008; MR-Egger: OR = 0.047, *p* = 0.008), Educational attainment (college completion) (IVW: OR = 0.047, *p* < 0.001; Weighted Median: OR = 0.021, *p* < 0.001), Eosinophil counts(IVW: OR = 0.751, *p* = 0.040),Basophil count (IVW: OR = 0.491, *p* = 0.039) (Fig. 2). Arm fat percentage (left) (*p*_pleiotropy_ = 0.007) and Arm fat percentage (right) (*p*_pleiotropy_ = 0.008) may exhibit pleiotropy effects, as indicated in Table 2.

**Table 2:** Variable risk factors for sporadic vestibular schwannoma

Exposure	SNPs	Inverse variance weighted		Weighted median		MR-Egger		*P*	*P*	*P*
		*P*	OR (95%CI)	*P*	OR (95%CI)	*P*	OR (95%CI)	MR-PRESSO	Heterogeneity	Pleiotropy
Related diseases										
Ovarian cancer	9	**0.028**	**1.484 (1.043–2.112)**	0.095	1.497 (0.932–2.405)	**0.039**	**3.131 (1.292–7.592)**	0.733	0.812	0.115
Leiomyoma of uterus (all cancers excluded)	15	**0.004**	**1.566 (1.152–2.130)**	0.051	1.453 (0.999–2.114)	0.227	1.454 (0.816–2.590)	0.226	0.187	0.766
Uterine fibroids	40	**0.016**	**1.348 (1.058–1.719)**	**0.046**	**1.450 (1.007–2.087)**	0.062	1.549 (0.991–2.420)	0.571	0.525	0.472
Stable angina pectoris	41	**0.004**	**0.681 (0.526–0.883)**	0.332	0.821 (0.551–1.223)	0.209	0.685 (0.383–1.225)	0.633	0.646	0.984
Alzheimer’s disease	18	**0.049**	**0.849 (0.721–0.999)**	0.382	0.913 (0.746–1.119)	0.292	0.879 (0.697–1.109)	0.340	0.265	0.674
Gastroesophageal reflux disease	66	**0.004**	**1.928 (1.238- 3.003)**	**0.049**	**1.919 (1.004- 3.671)**	0.789	1.402 (0.119–16.539)	0.666	0.669	0.798
Psoriatic and enteropathic arthropathies	4	**0.033**	**0.808 (0.665–0.983)**	0.139	0.835 (0.658–1.061)	0.697	0.896 (0.557–1.444)	0.545	0.481	0.684
Lifestyle habits										
Oily fish intake	51	**0.014**	**0.229 (0.071- 0.742)**	0.115	0.257 (0.047–1.390)	0.419	0.137 (0.001–16.311)	0.798	0.793	0.829
Dried fruit intake	35	**0.011**	**0.107 (0.019–0.594)**	**0.018**	**0.054 (0.005–0.600)**	0.264	98.150(0.036–267296.469)	0.759	0.724	0.092
Alcohol intake frequency	89	**0.008**	**2.023 (1.203–3.402)**	0.092	2.250 (0.877–5.774)	0.238	2.043 (0.629–6.628)	0.560	0.526	0.986
Morning/evening person (chronotype)	138	**0.008**	**2.463 (1.269–4.781)**	0.318	2.868 (1.096- 7.508)	0.836	1.251 (0.151–10.350)	0.776	0.742	0.509
Age at first birth	53	**0.008**	**0.712 (0.553–0.916)**	0.079	0.727 (0.509–1.038)	0.582	1.381 (0.441–4.318)	0.203	0.196	0.248
Age at first sexual intercourse	47	**0.017**	**0.381 (0.172–0.844)**	0.130	0.407 (0.127–1.303)	0.787	0.599 (0.015–24.149)	0.810	0.803	0.807
Nutritional status										
Arm fat percentage (left)	231	**0.012**	**1.975 (1.164–3.351)**	0.485	1.320 (0.606–2.877)	0.075	0.214 (0.039–1.162)	0.824	0.824	**0.007**
Arm fat percentage (right)	213	**0.011**	**2.014 (1.170–3.466)**	0.484	1.333 (0.596- 2.985)	0.079	0.210 (0.037–1.190)	0.912	0.894	**0.008**
Learning ability										
Fluid intelligence score	40	**0.013**	**0.671 (0.490–0.918)**	**0.034**	**0.638 (0.421–0.967)**	0.497	0.635 (0.174–2.326)	0.891	0.867	0.933
Cognitive performance	117	** < 0.001**	**0.409 (0.240–0.695)**	**0.003**	**0.317 (0.147–0.685)**	0.079	0.136 (0.015–1.233)	0.650	0.624	0.315
Qualifications: college or University degree	217	** < 0.001**	**0.070 (0.023–0.217)**	** < 0.001**	**0.050 (0.009–0.289)**	0.263	0.072 (0.001–7.183)	0.825	0.829	0.995
Qualifications: a levels/AS levels or equivalent	53	**0.011**	**0.055 (0.006–0.515)**	**0.006**	**0.011 (0–0.284)**	0.951	1.500 (0–625416.914)	0.393	0.384	0.613
Years of schooling	263	** < 0.001**	**0.348 (0.198–0.612)**	**0.008**	**0.317 (0.136–0.739)**	**0.008**	**0.047 (0.005–0.440)**	0.393	0.382	0.071
Educational attainment (college completion)	182	** < 0.001**	**0.047 (0.013–0.170)**	** < 0.001**	**0.021 (0.003–0.143)**	0.385	0.093 (0–19.334)	0.842	0.829	0.797
Laboratory indicators										
HDL cholesterol levels	305	**0.049**	**1.234 (1.001–1.522)**	0.559	1.121 (0.764–1.646)	0.110	1.267 (0.949–1.693)	0.154	0.160	0.796
Apolipoprotein A1 levels	249	**0.029**	**1.289 (1.026–1.619)**	0.491	1.156 (0.765–1.746)	0.178	1.251 (0.904–1.730)	0.586	0.566	0.799
Mean sphered cell volume	349	**0.035**	**1.291 (1.019–1.636)**	0.495	1.151 (0.769–1.721)	0.159	1.321 (0.898–1.945)	0.220	0.252	0.882
Eosinophil counts	317	**0.040**	**0.751 (0.572–0.987)**	0.704	0.913 (0.572–1.457)	0.388	0.800 (0.483–1.327)	0.203	0.199	0.773
Basophil count	75	**0.039**	**0.491 (0.250–0.966)**	0.660	0.799 (0.294–2.170)	0.119	0.330 (0.083–1.311)	0.384	0.377	0.518
Hemoglobin	307	**0.039**	**1.349 (1.015–1.792)**	0.109	1.503 (0.913–2.474)	**0.030**	**1.783 (1.060–3.002)**	0.663	0.628	0.201
Glycated haemoglobin HbA1c levels	305	**0.009**	**1.459 (1.099–1.939)**	0.234	1.303 (0.842–2.014)	0.256	1.013 (0.614–1.672)	0.073	0.070	0.084
Calcium levels	186	**0.009**	**1.652 (1.133–2.408)**	**0.032**	**1.934 (1.058–3.534)**	0.271	1.555 (0.710–3.404)	0.411	0.416	0.863

Bold *P*-values indicate statistical significance (*P* < 0.05)

Bold values in the OR (95% CI) column correspond to these significant *P*-values, highlighting their associated effect sizes and confidence intervals

## Discussion

This study elucidates the complex associations between diverse factors and the risk of developing sporadic vestibular schwannoma, a benign yet significant intracranial tumor. Findings encompass related diseases, lifestyle habits, nutritional status, learning abilities, and laboratory indicators, each offering unique insights into the etiology of disease and potential prevention strategies.

The analysis reveals intricate associations between sporadic vestibular schwannoma and a spectrum of related diseases, suggesting multifaceted biological pathways influence disease predisposition. The associations with ovarian cancer and uterine conditions, such as leiomyomas and fibroids, highlight the potential role of hormonal regulation and reproductive factors in schwannoma development. Hormonal dysregulation, particularly involving estrogen and progesterone, may influence tumor growth, as these hormones are known to affect cell proliferation in both reproductive tissues and schwannomas. Shared genetic variants and metabolic pathways may also contribute to these associations, particularly those related to estrogen signaling and inflammatory processes. For example, cases have been reported where chemotherapy for ovarian cancer led to regression of vestibular schwannoma, suggesting shared biological mechanisms such as BRCA1 mutations influencing tumor behavior in both conditions [37, 38]. Similarly, the association with stable angina pectoris introduces a cardiovascular dimension, suggesting that systemic vascular factors or endothelial dysfunction could play a role in tumor growth or its microenvironment, thus warranting further exploration of cardiovascular health in individuals affected by vestibular schwannoma. Additionally, the intriguing association with gastroesophageal reflux disease and autoimmune conditions, such as psoriatic and enteropathic arthropathies, supports the hypothesis of systemic inflammation, autonomic nervous system dysfunction, and immune system dysregulation underlying the etiology of sporadic vestibular schwannoma. Chronic inflammatory processes have been implicated in tumorigenesis, potentially creating a conducive environment for tumor development [39, 40]. These findings underscore the necessity of a multidisciplinary research approach, integrating hormonal, cardiovascular, gastrointestinal, immune, and metabolic perspectives to unravel the complex interactions contributing to sporadic vestibular schwannoma and to inform effective management strategies.

The analysis within the domain of lifestyle habits reveals meaningful associations with the risk of developing sporadic vestibular schwannoma, highlighting the impact of dietary intake, alcohol consumption, circadian rhythms, and reproductive timing on disease susceptibility. Consumption of oily fish, rich in omega-3 fatty acids [41], emerges as a protective factor against sporadic vestibular schwannoma, attributed to the anti-inflammatory [42] of omega-3 properties that counter inflammation, a potential contributor to tumor development [43]. Similarly, intake of dried fruits, dense in essential nutrients such as vitamins [44, 45], minerals [46], and antioxidants [47], appears to confer benefits, suggesting a nutrient-rich diet may play a role in maintaining neurological health and potentially thwarting tumor growth. In contrast, frequent alcohol intake has been identified as a risk enhancer for sporadic vestibular schwannoma, highlighting the negative effects of excessive alcohol consumption on health, potentially through exacerbating inflammation or other tumor-promoting mechanisms [48]. Studies have shown that alcohol consumption is associated with an increased risk of vestibular schwannoma, likely due to its influence on metabolic and immune responses that can promote tumorigenesis [49, 50]. Additionally, this study underscores the significance of circadian rhythm alignment, with individual chronotype preferences linked to disease risk. Disruptions in circadian rhythms may lead to hormonal imbalances, altered immune functions, and changes in metabolic processes, affecting the regulation of key hormones like cortisol and melatonin, both of which are essential for maintaining cellular health and reducing tumor development risk [51]. Moreover, reproductive timing factors, such as the age at first birth and first sexual intercourse, exhibit a protective association with the risk of sporadic vestibular schwannoma. Each additional year prior to these reproductive milestones is correlated with a decreased risk, hinting at a complex interplay between hormonal changes and the timing of reproductive events in modulating disease risk. This relationship underscores the importance of further exploration into the hormonal and biological mechanisms by which reproductive timing influences the risk of sporadic vestibular schwannoma [52]. Collectively, these findings underscore the importance of lifestyle and behavioral factors in the context of sporadic vestibular schwannoma risk. They advocate for a holistic approach to health promotion that incorporates dietary guidance, moderation in alcohol consumption, synchronization of circadian rhythms, and consideration of reproductive timing. Understanding the complex interactions between these lifestyle habits and sporadic vestibular schwannoma susceptibility is crucial for developing targeted prevention strategies and improving overall health outcomes.

The analysis of nutritional status, particularly focusing on arm fat percentage, underscores a significant association with the risk of sporadic vestibular schwannoma, linking higher fat percentages to increased susceptibility. This finding highlights the potential role of localized adiposity in the development of disease, suggesting fat distribution, rather than overall body fat, might play a crucial role in affecting physiological and metabolic states, potentially contributing to tumor growth [53]. This relationship necessitates a broader consideration of public health strategies and individual lifestyle adjustments for body composition management. Highlighting the importance of maintaining a healthy weight and balanced body fat distribution, the findings advocate for incorporating balanced nutrition and regular physical activity into daily routines as preventive measures against sporadic vestibular schwannoma. Furthermore, this observation calls for a deeper investigation into the specific biological processes by which localized body fat may predispose individuals to this condition. Understanding the precise mechanisms by which body fat distribution impacts the risk of sporadic vestibular schwannoma is essential for developing targeted interventions. Research focused on determining whether reducing localized fat deposits through specific lifestyle or medical interventions can offer protective benefits against developing sporadic vestibular schwannoma is particularly warranted. This approach contributes not only to the existing body of knowledge on the etiology of disease but also to the development of nuanced, effective prevention strategies tailored to individual risk profiles.

The examination of learning ability in relation to the risk of sporadic vestibular schwannoma reveals that enhanced cognitive performance and educational attainment are inversely associated with the development of disease. This relationship suggests that the advantages of education extend beyond socioeconomic benefits, potentially influencing biological factors that affect health. Higher levels of education are correlated with healthier lifestyles, improved health literacy, and better access to healthcare, factors that likely contribute to mitigating disease risk [54]. These insights underscore the critical importance of fostering cognitive development and supporting educational pursuits as key components of public health strategies for disease prevention. The findings advocate targeted interventions to bolster cognitive abilities and encourage educational advancement, highlighting the potential of such measures to decrease the incidence of sporadic vestibular schwannoma within the population. The strong correlation between educational attainment and diminished disease risk further highlights the need for policies that enhance educational access and opportunities, reinforcing the pivotal role of education in improving public health and reducing the burden of diseases such as sporadic vestibular schwannoma.

The analysis of laboratory indicators offers a nuanced understanding of the biochemical and hematological factors associated with the risk of sporadic vestibular schwannoma, revealing an intricate interplay among lipid metabolism, blood cell physiology, immune system activity, and mineral homeostasis in the pathogenesis of disease. The association of elevated HDL cholesterol and apolipoprotein A1 levels with an increased risk of sporadic vestibular schwannoma presents a paradox within the context of the role of lipid metabolism in tumorigenesis [55]. Despite their known protective effects against cardiovascular diseases, these lipid profiles may exert differential influences on tumor development, potentially via mechanisms affecting cell membrane dynamics, signaling pathways, or inflammatory responses, underscoring the complexity of lipid metabolism in cancer risk and highlighting the necessity for further investigation. Additionally, the link between increased mean sphered cell volume and sporadic vestibular schwannoma risk emphasizes the importance of erythrocyte physiology, as changes in red blood cell size and function could influence oxygen delivery, blood viscosity, and microvascular circulation within the tumor microenvironment [56]. Conversely, elevated eosinophil and basophil counts, associated with decreased risk, underscore the role of immune system in modulating tumor risk, possibly through immune surveillance and the impact of inflammatory milieu on tumor growth and progression [57]. Moreover, the associations of higher hemoglobin [58] and HbA1c levels [59] with increased risk may indicate the influence of conditions such as polycythemia or diabetes on tumorigenesis, via increased blood viscosity and chronic hyperglycemia-induced oxidative stress. Lastly, the connection between higher calcium [60] levels and elevated risk emphasizes the vital role of calcium in cancer-relevant cellular processes, suggesting dysregulated calcium homeostasis may be implicated in the pathogenesis of sporadic vestibular schwannoma, affecting growth and communication pathways. The examination of laboratory indicators illuminates potential biological mechanisms underlying sporadic vestibular schwannoma risk, highlighting the pivotal roles of lipid profiles, blood cell characteristics, immune responses, and mineral balance. These findings emphasize the need for a comprehensive approach to understand sporadic vestibular schwannoma, incorporating biochemical and physiological markers into risk assessment and disease management strategies. Further research is imperative to elucidate the mechanisms through which these laboratory indicators influence sporadic vestibular schwannoma development, providing potential targets for intervention and novel insights into the etiology of disease.

The identified risk and protective factors from this study provide valuable insights for clinical application, particularly in the realms of prevention and diagnosis of sporadic vestibular schwannoma. Conditions such as ovarian cancer, uterine fibroids, and GERD highlight the need for more focused monitoring of individuals with these comorbidities. Lifestyle factors, such as alcohol consumption and circadian rhythm disruption, point to the potential benefits of behavioral interventions aimed at reducing tumor risk. Additionally, the role of metabolic markers (e.g., HDL cholesterol, HbA1c) and educational attainment underscores the importance of integrating both biological and social factors into comprehensive risk assessment models. These findings advocate for a multidisciplinary approach in the early identification and prevention of vestibular schwannoma, through personalized screening protocols and lifestyle modifications tailored to the individual’s risk profile.

This study employs a Two-Sample MR approach to uncover genetic risk factors for sporadic vestibular schwannoma, yet it is constrained by several limitations. Primarily, the reliance on SNPs as IVs might not capture the full spectrum of genetic variations, potentially limiting the generalizability of our findings. Moreover, the study’s focus on populations of European descent restricts the applicability of the results across diverse racial and ethnic backgrounds, which may not reflect the genetic risk factors pertinent to those groups. Furthermore, the scope of the datasets analyzed and the statistical power might not encompass all relevant genetic risk factors, indicating that some genuine associations could remain undetected. Additionally, environmental and lifestyle factors, such as diet or exposure to environmental toxins, were not considered in our analysis, which could interact with genetic predispositions and influence disease risk. Future research should aim to include a wider array of genetic variations, expand the study population to include more diverse racial and ethnic groups, and incorporate functional studies to validate these findings in larger cohorts and explore the biological mechanisms suggested by our results.

## Conclusions

**Fig. 3:**
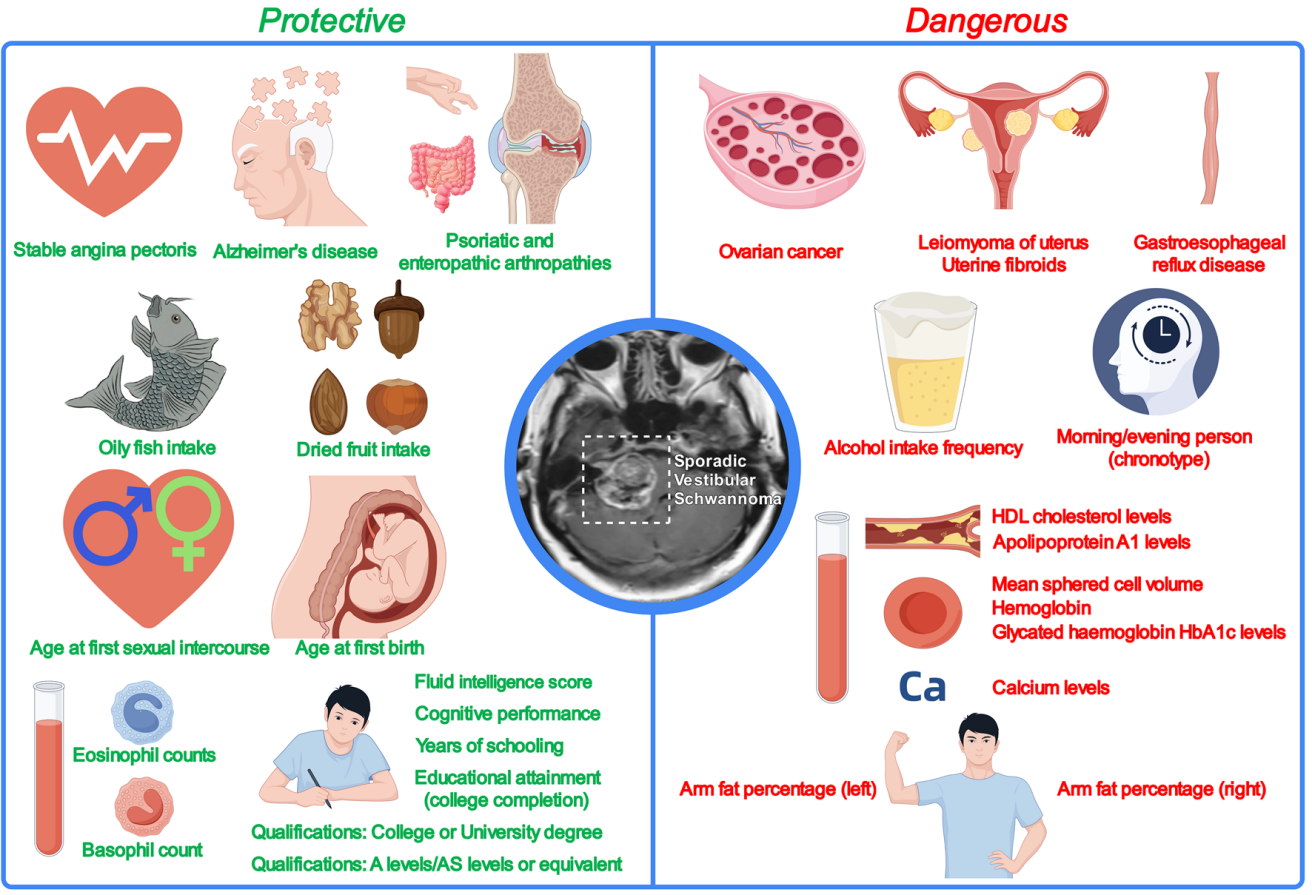
Genetic insights into vestibular schwannoma risk factors through MR. Copyright information: FigDraw, ID UYARTbecc3

This study provides a comprehensive analysis of factors associated with sporadic vestibular schwannoma risk (Fig. 3). These insights, particularly alcohol consumption, ovarian cancer, and uterine fibroids, not only enhance the understanding of the etiology of the disease but also inform potential prevention strategies and underscore the importance of a multidisciplinary approach to research and public health initiatives targeting sporadic vestibular schwannoma. Further investigation into the underlying mechanisms of these associations is essential for developing effective interventions and improving patient outcomes.

## Data Availability

GWAS summary data of Ovarian cancer, Oily fish intake, Dried fruit intake, Qualifications: College or University degree, Alcohol intake frequency, Morning/evening person (chronotype), Alzheimer's disease, Qualifications: A levels/AS levels or equivalent, Arm fat percentage (left), Arm fat percentage (right), Fluid intelligence score, Years of schooling, Cognitive performance, Leiomyoma of uterus (all cancers excluded), Psoriatic and enteropathic arthropathies were downloaded from OCAC, MRC-IEU, ADGC, EADI, CHARGE, GERAD/PERADES, Neale Lab, SSGAC, FinnGen. For Uterine fibroids, Stable angina pectoris, Glycated haemoglobin HbA1c levels, Calcium levels, Basophil count, Gastroesophageal reflux disease, Educational attainment (college completion), HDL cholesterol levels, Apolipoprotein A1 levels, Mean sphered cell volume, Eosinophil counts, Hemoglobin, Age at first birth, Age at first sexual intercourse, sporadic vestibular schwannoma, the GWAS summary data were selected from associated GWAS studies.
